# A system-level analysis of challenges and strategies for scaling artificial intelligence in healthcare: A qualitative study of the NHS AI lab

**DOI:** 10.1177/20552076261464270

**Published:** 2026-08-10

**Authors:** Hajar Mozaffar, Robin Williams, Stuart Anderson, Kathrin Cresswell

**Affiliations:** 1 Business School, 3124 University of Edinburgh, Edinburgh, UK; 2 Institute for the Study of Science, Technology and Innovation, 3124 University of Edinburgh, Edinburgh, UK; 3 School of Informatics, 3124 University of Edinburgh, Edinburgh, UK; 4 Usher Institute, 3124 University of Edinburgh, Edinburgh, UK

**Keywords:** artificial intelligence in healthcare, scaling, pilot-to-scale gap, adoption, system-level mechanisms, health system transformation

## Abstract

**Objective:**

Despite Artificial Intelligence’s (AI) promise in healthcare, achieving widespread and lasting adoption remains a global challenge. While pilot studies show positive impacts, the reasons for limited uptake in practice are underexplored. This paper examines challenges hindering AI scaling in healthcare and develops a system-level explanation of why these challenges persist, alongside strategies to address them.

**Methods:**

As part of a wider evaluation of England’s National Health Service (NHS) AI Lab, we conducted in-depth semi-structured interviews, documentary analysis, and observations of related meetings and events between April 2024 and April 2025. Data were analyzed using reflexive thematic analysis, followed by a higher-level conceptual synthesis.

**Results:**

We identified 15 interrelated challenges spanning market forces, healthcare adopter dynamics, and macro-environment challenges. Challenges included unclear pathways from pilots to adoption, fragmented regulatory and evaluation processes, limited funding for scaling, procurement and organizational capability gaps, uneven digital maturity, unclear partnership models, uncertain returns on investment, and difficulties navigating fragmented healthcare systems. Rather than operating as discrete issues, these challenges reflect four underlying and interdependent system-level mechanisms: fragmented innovation pathways, fragmented governance and lack of system orchestration, organizational transformation deficits, and market and incentive misalignment. Together, these mechanisms help explain why AI innovations frequently struggle to progress from pilot experimentation to routine and system-wide adoption.

**Conclusions:**

Our findings shift the focus from isolated challenges to the system-level dynamics through which challenges to AI scaling emerge, interact, and persist. The study contributes an empirically grounded explanation of the pilot-to-scale gap and highlights how governance, organizational, market, and innovation dynamics jointly constrain scaling. Building on these findings, we propose a set of coordinated strategic responses that emphasize system orchestration, organizational transformation, coherent innovation pathways, and continuous evaluation across the AI lifecycle.

## Introduction

Artificial intelligence (AI) has transformative potential for the healthcare sector,^1^ offering innovative solutions across various specialties and functions. Successful applications have demonstrated substantial potential to enhance diagnostic accuracy (for instance by improving patient triage in emergency departments),^2^ streamline administrative processes (for instance forecasting of demand and patient arrivals),^3^ and improve patient outcomes.^4,5^ Despite being a global priority, progress in scaling effective AI interventions in health and social care has been slow and uneven. . Scaling involves going beyond successful pilot implementations of AI in the healthcare setting, replicating AI interventions across sites, and systematic support for widespread implementation.^6,7^

Recent studies have attempted to identify some of the challenges to scaling of AI in healthcare. For instance multiple studies highlight lack of acceptance and trust, lack of awareness about AI applications, maintaining data privacy, biases in data sets, high adoption costs, and organizational barriers to achieving the widespread and lasting adoption of AI innovation in healthcare.^8,9^ While insightful, the majority of these studies provide only a partial picture of the challenges by mainly focusing on adopter organizations and individual users. Some studies also identify other *generic* challenges such as immaturity of regulations or lack of clear information governance as barriers to scaling of AI in the healthcare sector.^10^ While such studies offer a valuable starting point, they often lack deeper theoretical insight into factors - beyond organizational and regulatory challenges - that explain why many AI projects in healthcare fail to move beyond the pilot stage and scale into routine practice across the healthcare system. Scaling AI in health and social care depends not on isolated organizational factors but on the dynamic interplay between different stakeholders and levels.^11^ The variety of potential challenges and interactions between them increase the difficulties of achieving successful adoption and scale. Understanding these interactions requires interdisciplinary, context-sensitive approaches that move beyond traditional evaluation methods to capture the evolving relationship between different organizations and their macro-environment conditions. This paper aims to unpack the multitude of system-level challenges that hinder AI scaling in the health sector, examining the interplay between market forces, inter-organizational adoption dynamics, and macro-environment challenges. In this study we address the following research questions:

• What challenges hinder the scaling of AI innovations in healthcare systems?
• How do these challenges reflect underlying, interrelated system-level mechanisms that constrain the transition from pilot experimentation to regional and system-wide adoption?
• What strategic responses can address these mechanisms to enable sustainable, system-wide scaling of AI in healthcare?

In this paper, we use the term scaling to refer to the process through which AI innovations move beyond localized pilots or early implementation sites toward broader, sustained, and system-wide adoption across healthcare settings. Though this study we intend to provide insights into how AI innovations can navigate the complex landscape of healthcare and transition from promising ideas and pilots to widespread adoption and use in practice.

We draw on our evaluation of the United Kingdom’s National Health Service (NHS) Artificial Intelligence Laboratory (hereafter AI Lab), established in UK in 2019 as a major government initiative, with an initial investment of £250 million, aimed to facilitate and support the development and deployment of AI technologies at scale, across the health and care system in the UK.

## Material and methods

This study was a part of a larger program of work into the evaluation of AI Lab in England. Drawing on recent recommendations on the importance of conducting rigorous qualitative research in health informatics that explores the views of different stakeholders and locales,^12^ we adopt a multi-level approach^13^ in studying the challenges that hinder scaling of AI in the health sector by examining the views of policy-makers, technology suppliers, and technology-adopters.

### Sampling

We used purposive sampling to capture diverse stakeholder perspectives on AI Lab activities, including developers, implementers, managers, decision-makers, regulators, evaluators, academics, and professional bodies. The AI Lab leadership, who commissioned the study, provided an initial stakeholder list. To mitigate the risk of potential bias, we expanded the sample through the research team’s professional networks and snowball sampling, actively seeking perspectives beyond those initially identified by the programme team. Given that the study was commissioned by NHS England and focused on the NHS AI Lab, we also took steps to protect analytic independence. The research team operated independently in data collection, analysis, and interpretation. Participants were informed that the study was conducted for evaluation and research purposes, and that their responses would be treated confidentially.

In addition to interviews, we conducted case-based analysis of selected projects funded through the NHS AI Lab Artificial Intelligence in Health and Care Award, which supported 86 AI pilot projects across different stages of development.^14^ The Award is structured into four phases corresponding to technology readiness, ranging from early feasibility testing to system adoption within the NHS. Early phases (Phases 1–2) focus primarily on feasibility testing and early prototype development. Phase 3 supports real-world evaluation of AI technologies in clinical or operational settings, while Phase 4 supports technologies that have demonstrated feasibility and are undergoing broader implementation and evaluation across NHS organizations to assess readiness for wider adoption and scaling.

Our 10 case studies were drawn from Phase 3 and Phase 4 projects, as these represent technologies that had progressed beyond early development and were being deployed in real-world healthcare environments. Including both phases allowed us to examine scaling challenges across two critical stages of the innovation pathway: late-stage evaluation (Phase 3) and early system adoption and expansion (Phase 4). These stages are particularly appropriate for investigating challenges to scale because they involve interaction with healthcare delivery systems, regulatory processes, procurement mechanisms, and organizational workflows.

The case studies included AI technologies addressing a range of clinical and operational use cases supported through the NHS AI Lab programme. Consistent with the distribution of technologies reported in the programme evaluation, these applications primarily focused on screening and diagnostic support (e.g., analysis of medical imaging and diagnostic tests), risk prediction and prognostic modelling, clinical triage and care pathway prioritization, and decision-support tools designed to assist clinicians in treatment planning or workflow management. Some projects also targeted operational and administrative processes, such as improving documentation, resource allocation, or service coordination within healthcare organizations. Together, these applications reflect the broader portfolio of AI technologies supported by the NHS AI Lab.

While focusing on Phase 3 and Phase 4 projects enabled in-depth examination of scaling challenges under real-world implementation conditions, this sampling strategy may underrepresent projects that did not progress beyond early development phases. To mitigate this limitation, we complemented the case study analysis with interviews involving stakeholders across the broader AI innovation ecosystem, including developers, policymakers, regulators, implementers, and evaluators involved at different stages of the AI development and adoption pathway.^15^

### Data collection

Data collection took place between June 2024 and April 2025 by four authors. We conducted documentary analysis, in-depth semi-structured interviews, and observation of meetings and events related to NHS AI Lab. Table 1 illustrates the different components of the AI Lab.

**Table 1.** Different components of the AI Lab.

Component	Description
Infrastructures	This component aimed to focus on development of the National COVID-19 Chest Imaging Database and AI Deployment Platform.
Knowledge sharing and community building	This component aimed to focus on training and upskilling of staff, cultural change and building confidence in developing and implementing AI, and nurturing communities of practice of AI practitioners in health and care.
Innovation, research, and evidence	This component presented the AI in Health and Care Awards: 86 projects at different phases of adoption (technical and clinical feasibility of the concept, prototyping and generating early safety and efficiency data, first real-world testing in health and social care settings, supporting adoption for technologies with market authorization but insufficient evidence for large scale commissioning or deployment).
Skunkworks projects	This component examined proof-of-concept projects using agile development.
Governance and ethics	This component focused on developing AI policy and regulation ecosystem, and the AI ethics initiative which aimed to build confidence in AI.

**Documentary analysis:** We reviewed 1021 AI Lab documents including business cases, lessons learned reports, monthly reports, meeting minutes, and evaluation data (including existing analyzes, benefits maps, project reports, and existing evidence of outcomes and impact documented in logic models) collected across all components of the AI Lab. These documents were collected across all components of the AI Lab and were used to contextualize interview accounts, trace programme-level decision-making, identify documented barriers and enabling conditions, and compare stated objectives with reported implementation and scaling outcomes.

**Interviews:** We collected data through semi-structured interviews with 85 individuals - including NHS England staff, academics, suppliers, Department of Health and Social Care (DHSC) staff, previous AI Lab staff, and regulators. Table 2 demonstrates the participants details. The interviews explored experiences, perceived and realized outcomes over time, challenges and ways to address them, and potential lessons for future programs. Box 1 presents the interview guide. For the purposes of this paper, we tailored the interview guide to participants involved in scaling to ask questions about a) the challenges and barriers to scaling of AI; and b) strategies to overcome these challenges. Interviews lasted between 30 to 90 minutes, with an approximate average of an hour. Participants gave written informed consent to participate in the interviews, which were conducted online via Microsoft Teams. The interviews were audio-recorded and transcribed verbatim using the built-in transcription software.

**Box 1. Overall Interview Guide**

• Exploration of involvement in the AI Lab?
• Opinions of the AI Lab as an initiative including initial vision and to what extent this has been realized, nature of programme activities and innovations, expected and realized benefits
• Intended and unanticipated consequences, positive and negative
• Challenges and potential ways to address these
• Differences between AI Lab components
• Lessons learned and implications for future initiatives

**Table 2.** Participant details (5 participants were interviewed twice).

Participant role	Number of interviewees
NHSE Staff	22
Academics	15
Suppliers	14
Department of Health and Social Care	8
Previous AI Lab Staff	6
Regulators	3
Others (advisors, innovation intermediaries, implementation partners, evaluation partners, professional bodies)	17

**Observation:** We also collected data through observation of 12 project progress meetings, AI Lab board meetings and workshops held by the AI lab. In the observations, we purposefully explored and collected observation notes with respect to a) what is hindering scaling beyond pilot developments; b) strategies discussed to overcome the scaling challenges. Observational data were used to capture real-time discussions and decision-making processes, and to triangulate themes emerging from interviews and documentary analysis.

Data collection continued until the research team judged that there was sufficient depth, diversity, and recurrence in the data to address the study aims and support the development of the analytical themes. Rather than treating saturation as the point at which no new data could emerge, we used the concept of analytic sufficiency to indicate that subsequent interviews and observations were reinforcing, rather than substantially altering, the emerging interpretation.

### Data analysis

We drew on the multi-level analytical approach to capture views from adopters and suppliers of AI innovations, as well as those involved in shaping the macro-environment (e.g., policymakers and AI Lab decision-makers).^13^ This This enabled us to move beyond a single implementation or adopter perspective and examine how challenges to scaling emerge across interconnected levels of the healthcare innovation ecosystem.

Data were analyzed using reflexive thematic analysis.^15^ Data analysis was conducted in four iterative phases.

### Phase 1: Data familiarization and initial coding

The lead researcher (HM) led the analysis and used NVivo 14 to organize and integrate data from interviews, observations, and documents. HM conducted inductive open coding across all data sources to capture the breadth and diversity of stakeholder experiences. This stage generated a comprehensive set of initial codes reflecting key issues, tensions, and perspectives related to AI adoption and scaling.

### Phase 2: Development and refinement of themes

In the second phase, initial codes were iteratively grouped and refined into higher-order themes. This process involved identifying patterns of meaning across the dataset and comparing similarities and differences between stakeholder groups (e.g., adopters, suppliers, and policy-makers). Coding and theme development were primarily conducted by HM, with KC, RW, and SA reviewing selected transcripts, codes, and emerging themes. Regular discussion meetings were held to critically reflect on interpretations, resolve differences, and iteratively refine the coding structure and thematic framework. This process provided ongoing consistency checking and analytical rigor, consistent with a reflexive thematic analysis approach, which emphasizes interpretive depth over statistical inter-coder reliability. Through this analysis, we identified patterns of meaning, or emerging issues, which were then categorized into themes – presented in column 3 of Table 3. We focused on challenges that extend beyond individual organizations. This involved comparing similarities and differences across these perspectives, as well as exploring potential underlying reasons for any discrepancies. To enhance rigor and reduce risk of bias, we employed data triangulation by integrating insights across interviews, observational field notes, and documentary sources.

**Table 3.** Illustrative examples of moving from data extracts to themes and categories.

Data extract (Illustrative quote)	Initial codes (Phase 1: Open coding)	Higher-orderTheme (Phase 2)	Organizing category
“The AI Lab was more interested in early-stage research rather than pulling things through into operational impact.”	Focus on early-stage innovation Lack of operationalization Weak scaling focus	Limited focus on funding AI scaling	Macro-environment
“Innovation valley of death… great ideas but not translated into sustainable capability.”	Discontinuity after pilots Lack of sustainability Failure to scale	Unclear pathway from pilot implementation to widespread adoption	Macro-environment
“There was no docking place for them to go next… community getting frustrated.”	No pathway forward Lack of integration route Supplier frustration	Unclear pathway from pilot implementation to widespread adoption	Technology supply
“My view is that is AI a sticking plaster to a badly organized and designed service or process; it is just the wrong approach…”	AI as superficial fix Need for deeper service redesign Transformation beyond implementation	Need for rethinking healthcare service design and delivery	Adopter
“You’ve got to have all of these things lined up… commercial, procurement, finance…”	Lack of coordination Fragmented decision-making Complex governance	Poor alignment between regulatory processes and the dynamics of AI innovation	Macro-environment
“Struggles to navigate the fragmented healthcare landscape.”	System complexity Barriers to entry Navigation challenges	Struggles to navigate the fragmented and complex healthcare landscape	Technology supply

Following theme development, themes were further organized into organizing three categories: (1) challenges related to healthcare adopters, (2) challenges related to developers and suppliers of AI systems, and (3) challenges associated with the macro-environment. These three categories were used as an intermediate organizing structure and reflect the primary domains within which challenges were initiated. However, subsequent analysis showed that the challenges cut across these categories and interacted dynamically, leading to the development of the higher-order mechanism-level synthesis presented in Phase 3.Table 3, illustrates examples of how we have moved from data extraction to coding and theme development. Many of the identified challenges were raised across multiple stakeholder groups, highlighting their cross-cutting nature and reinforcing their character as system-level concerns.

### Phase 3: Conceptual abstraction and system-level synthesis

In the third phase, we moved beyond descriptive thematic categorization to develop a higher-order analytical interpretation of the findings. While the 15 challenges were initially organized across macro-environment, organizational (adopter), and technology supply domains (as illustrated in Table 4), further analysis showed that these challenges did not operate as discrete or independent barriers, but clustered into recurring and interrelated patterns across the dataset. Instead, through iterative analytical synthesis, they were interpreted as reflecting four underlying and mutually reinforcing system-level mechanisms: (1) fragmented and project-based innovation pathways (“pilot-to-scale gap”), (2) fragmented governance and lack of system orchestration, (3) organizational transformation deficits, and (4) market and incentive misalignment. The four mechanisms did not emerge as a separate coding stage, but through an iterative process of analytical synthesis in which we examined how the identified challenges clustered, interacted, and reinforced one another across macro-environment, organizational, and market domains. The four mechanisms were selected because they provided the most conceptually coherent explanation for the recurring patterns observed across the dataset, while preserving the interdependencies between challenges. Alternative conceptual groupings were considered during analysis; however, these either reproduced descriptive categories or failed to adequately capture the cross-level dynamics observed across stakeholder groups and data sources.

**Table 4.** Descriptive challenges identified across system domains.

Organizing category	Challenges to scaling
Macro-environment challenges	• Unclear pathway from pilot implementation to widespread adoption; • Differences across adopting countries (roles, healthcare funding, etc.); • Lack of system-view of change; • Unclear mechanisms to systematically assess and approve AI adoption; • Limited focus on funding AI scaling; • Broader economic and political drivers; • Poor alignment between regulatory processes and the dynamics of AI innovation;
Adopter Challenges	• Limited procurement expertise by healthcare provider organizations; • Need for rethinking healthcare service design and delivery; • Differences in levels of digital and data maturity; • Absence of shared digital platforms;
Technology Supply challenges	• Unclear partnership models; • Lack of clear return on investment; • Struggles to navigate the fragmented and complex healthcare landscape; • Long lag time between product launch and income;

This conceptual abstraction involved iteratively examining relationships between challenges, comparing patterns across stakeholder perspectives and data sources, and mapping these recurring interdependencies onto the four higher-order mechanisms. The analysis highlighted how these mechanisms interact across levels, for example, how fragmented governance contributes to unclear innovation pathways, how weak pathways exacerbate market uncertainty, and how organizational capability gaps further constrain implementation. Together, these interdependencies create a self-reinforcing negative feedback loop that limits the transition from pilot experimentation to system-wide adoption.

This stage moved beyond descriptive thematic categorization toward interpretive explanation, consistent with reflexive thematic analysis approaches that support conceptual abstraction and theory development from qualitative data.

The four mechanisms were supported by evidence from multiple stakeholder groups and data sources. *Fragmented innovation pathways* (M1) were most strongly reflected in discussions with suppliers, adopters (NHSE Staff), and policymakers regarding difficulties moving beyond pilot funding and implementation. *Fragmented governance and lack of system orchestration* (M2) emerged particularly from regulators, policymakers, NHSE staff, and suppliers describing fragmented responsibilities and approval processes. *Organizational transformation deficits* (M3) were most evident in accounts from adopters and implementation stakeholders as well as academics involved in different aspects of the NHS AI lab, concerning service redesign, procurement capability, and digital maturity. *Market and incentive misalignment* (M4) was especially prominent in supplier accounts relating to investment, commercialization, procurement, and return on investment. All four mechanisms were supported by interview data and were further corroborated through documentary analysis and observational field notes, providing triangulated evidence across data sources.

Illustrative quotes from interviews and observational field notes were selected after themes were finalized to support transparency and demonstrate how interpretations were grounded in the data.

We organized the presentation of this study in accordance with the Consolidated Criteria for Reporting Qualitative Research (COREQ) guidelines to enhance transparency, rigor and completeness.^16^

### Phase 4: Strategic response development

In a final interpretive phase, we moved beyond explanation of the identified mechanisms to consider their practical implications for addressing challenges to AI scaling. Drawing on the four system-level mechanisms, the underlying empirical challenges, and relevant literature on innovation, implementation, and digital transformation, we developed a set of strategic responses intended to address the recurring dynamics identified in the analysis. These strategic responses did not emerge directly from coding or thematic development and should not be interpreted as empirical findings. Rather, they represent higher-order analytical and practical implications derived through engagement between the empirical findings and existing theory and evidence. The resulting strategies are aligned with specific mechanisms while also recognizing their interdependencies across the wider system. These strategies are presented and discussed in the Discussion section.

## Results: System-level challenges in scaling artificial intelligence

Analysis identified 15 interrelated challenges that constrained the scaling of AI innovations beyond pilot implementation. While these challenges initially emerged across different parts of the healthcare ecosystem - including policy and regulatory environments, healthcare provider organizations, and technology suppliers, as presented in Table 4 - further analysis showed that they rarely operated as isolated barriers. Instead, stakeholders consistently described interconnected patterns of constraints that reinforced one another across organizational, market, and system levels. Through iterative analytical synthesis, the 15 challenges were interpreted as reflecting four interrelated system-level mechanisms that help explain why AI innovations frequently struggle to progress from pilot experimentation to sustained, large-scale adoption. The findings below are organized around these mechanisms and illustrate how they were experienced and described by stakeholders across the AI innovation ecosystem.

### Fragmented and project-based innovation pathways (“pilot-to-scale gap”)

The first mechanism concerns the absence of coherent pathways through which AI innovations can move from early experimentation to routine use at scale. Stakeholders repeatedly described innovation processes that were heavily oriented towards pilot projects, proof-of-concepts, and short-term funding cycles, but lacked clear routes for broader deployment, commercialization, and long-term sustainability. Challenges associated with scaling funding, evidence generation, procurement, approval processes, and cross-organizational adoption frequently converged around this issue. As a result, many promising innovations demonstrated technical feasibility but struggled to progress beyond isolated implementations, creating what participants frequently described as a recurring pilot-to-scale gap.

Multiple stakeholders highlighted that while AI technologies may eventually achieve market authorization based on quality, safety, and cost-effectiveness, the **pathway from pilot implementation to widespread adoption remains unclear**. Uncertainty around intellectual property (IP) ownership, commercialization models, and contracting continues to complicate scaling efforts. Despite DHSC having long-standing ambitions for cost recovery and commercialization, along with policies and mechanisms to support it, there remains a lack of clarity about key issues, such as whether to pursue share ownership, license fees or cut price access, and limited understanding of how these choices might impact the market. The risk of market distortion and the challenge of ensuring fair competition further complicate policy decisions on AI scaling.

“You couldn't go to market until that commissioned agreement was signed… So I think, yeah, realistically, it'll be two years or so, but the time it actually gets signed on the dotted line. So that's been extremely challenging and the kind of commercial hoops that we would have to jump through weren't made aware. We weren't made aware of that up front or at the start of the project.” (Supplier)

Whilst organizations like Gartner provide market insights into digital innovations in commerce there is no clearly defined body responsible for integrated and system-wide assessment of AI technologies in the healthcare sector. In the UK, this role is distributed across multiple institutions. NICE and the Medicines and Healthcare products Regulatory Agency (MHRA), both independent organizations sponsored by the Department of Health and Social Care (DHSC), play critical roles in evaluation and regulation.

NICE undertakes horizon scanning, technology assessment, guideline development, and advice to companies on evidence generation, while MHRA is responsible for ensuring the safety and regulatory compliance of medical technologies. However, despite these established roles, stakeholders described a **lack of coherent and coordinated pathways for assessing, approving, and adopting AI technologies at scale**. This reflects a broader institutional landscape in which responsibilities for innovation, regulation, procurement, and adoption are distributed across multiple actors with differing priorities. In particular, tensions between health-system objectives led by DHSC and innovation-driven agendas, such as those associated with the Department for Science, Innovation and Technology (DSIT), create competing pressures around risk, evidence, and speed of adoption. Within this context, the NHS operates as a major procurer, adopter, and enabler of innovation, which can further complicate decision-making and alignment across the system. While NICE plays a central role in ensuring that technologies are clinically effective and cost-effective, its emphasis on robust evidence, which tends to be time-consuming and resource-intensive to generate, can introduce inertia in the context of rapidly evolving AI technologies. At the same time, not all stakeholders involved in AI innovation programs were fully aware of, or engaged with, these evaluation processes, contributing to fragmentation and uncertainty. Recognizing these challenges, the NHS AI Lab supported efforts to adapt the NICE Evidence Standards Framework for Digital Health Technologies to better account for the characteristics of AI, highlighting ongoing attempts to align evaluation approaches with the pace and nature of innovation.

While the multiplicity and diversity of public funding schemes benefit various actors in the ecosystem, the **lack of coherence between funding mechanisms and the limited strategic focus on scaling** create uncertainty for developers and healthcare providers. This is especially problematic for start-ups, which, without sustained support beyond early development, risk falling into the “valley of death,” where promising innovations fail to progress due to a lack of long-term investment and system readiness.

“I think that with the AI initiative, scale has been an afterthought… it [scaling] hasn't showcased on enough… we know that if we're gonna do really effective scale across the system, we need to be thinking about scale before we even start just hasn't been the focus I think on scale.” (Advisor)

AI vendors, particularly startups and small enterprises, **struggle with the long lag between investing in development of AI innovations and securing reimbursement through sales**. Developing AI solutions for healthcare requires sustained investment, and this lag is often longer and more demanding than in other sectors due to the need for evidence generation to meet regulatory and evaluation requirements. In the UK context, this includes demonstrating safety and effectiveness for the MHRA, alongside building an economic case aligned with the NICE framework. However, many funding opportunities focus on relatively low cost, high risk early-stage innovation rather than long-term commercialization and scaling. Without adequate financial support, vendors face challenges in refining, validating, and deploying AI at scale.

“But clearly funding is quite challenging… The AI lab was more interested in the early-stage research rather than pulling things through into an operational impact. So probably finished too soon, and I'm sure you're aware of the term sort of innovation valley of death. Where you get some great ideas you can show real capability, but actually being able to put it through into a sustainable capability, which frankly is what is the most important thing.” (Intermediary)

### Fragmented governance and lack of system orchestration

The second mechanism reflects fragmentation in the governance, coordination, and oversight of AI innovation across the healthcare system. Stakeholders highlighted uncertainty regarding how responsibilities were distributed across multiple organizations with different objectives, timelines, and incentives, creating difficulties in aligning innovation priorities with healthcare delivery needs. Rather than a single barrier, participants described a broader absence of systemic orchestration capable of coordinating activities across regulators, policymakers, healthcare organizations, suppliers, and evaluators. This fragmentation often resulted in duplication of effort, uncertainty, and delays in decision-making.

The UK healthcare market, with its dominant player the NHS, presents both opportunities and challenges for AI vendors. While its national structure offers a unified consumer market, the complexity of procurement, regulation, and stakeholder engagement can deter companies from long-term investment. Additionally, **differences between private and public healthcare systems across countries** create varying incentives for AI vendors, making it difficult to develop a scalable approach that fits both UK and international markets. For instance the NHS’s procurement approach, including its standard government contracts and payment models was deemed *“too complex compared to US” (Supplier)*, making UK to be less attractive to AI vendors. Rigid pricing structures and lengthy procurement processes defined by the NHS may not align with the needs of AI companies, particularly those that rely on subscription-based or outcome-driven pricing models. As a result, some vendors opt to focus on markets with more flexible payment mechanisms.

“There hasn't been a commercial agreement that has been decided. So financially we haven't really seen a huge benefit just because of the kind of restrictions that were placed on us [by NHS]” (Supplier)

Another important challenge that was identified by our study participants, was a **lack of system-view** to how AI can enhance health outcomes or improve system efficiency. Instead, there was a tendency to focus on automating existing tasks within current workflows and regulatory frameworks, rather than pursuing broader service or system transformation. This led to narrow, use-specific implementations with limited potential for scaling, while the promise of AI lies in its transformational potential.

“And it's been very focused on individual clinically led initiative… it hasn't been focused on the big picture and what are we trying to achieve here… because it's the idea that if you do a load of like individual clinically led projects that somehow and they'll all join together and make a big scale change the NHS, and they won't.” (Advisor)

The commercialization of AI in healthcare is influenced by **broader economic and political drivers**, including lobbying by commercial technology firms and international trade agreements, such as those involving the US, or change of policy-makers and their visions on AI. Some of these forces prioritize technology-driven innovation over system-level needs, resulting in potential misalignment with actual healthcare priorities - for instance development of an innovative AI tool that reaches out to patient who may miss appointments, but does not impact their behavior. Cross-national differences further influence these dynamics. In the US, regulatory approval processes led by the Food and Drug Administration (FDA) are often perceived as more permissive, contributing to a faster-moving innovation environment, while fee-for-service models and specialized providers such as RadNet create clearer commercial incentives and support scaling. In contrast, the UK operates within a more centrally coordinated, publicly funded system, where adoption is shaped by cost-effectiveness requirements and system-wide priorities. Within this context, changes in political leadership, such as changes in ministers and their policy direction, can influence, slow, or redirect AI adoption and implementation processes.

“There was political involvement, so ministers have to sign things off. We also had things like elections happening in that period and also there's reasons for it, but the result is for a programme which wants to accelerate adoption of AI in the market. This unfortunately did quite a good job of slowing it down for us on this product.” (Supplier)

AI developers must navigate evolving regulatory frameworks that can change mid-development, creating uncertainty for businesses and healthcare providers. This challenge is compounded by the fact that AI, particularly statistical and data-driven models, does not align easily with traditional approaches to software safety and validation, which were designed for more deterministic systems. As regulatory frameworks rapidly evolve to accommodate these differences, a **poor alignment between regulatory processes and the dynamics of AI innovation** complicates long-term planning and increases the risk of non-compliance, further hindering AI adoption.

“It was very challenging because the regulatory landscape has also been changing over that time period” (Programme Team)

### Organizational transformation deficit

The third mechanism concerns the tendency to view AI primarily as a technological intervention rather than as part of a broader organizational transformation process. Stakeholders consistently emphasized that successful scaling required changes to workflows, service models, governance arrangements, professional practices, and organizational capabilities. However, many implementation efforts focused on introducing AI tools into existing structures rather than redesigning services around new possibilities created by AI. Differences in digital maturity, data infrastructure, procurement capabilities, evaluation capacity, and organizational readiness further reinforced these challenges. Consequently, scaling was often constrained not by technical limitations alone, but by the difficulty of achieving wider organizational and service transformation.

A key barrier to scaling AI in UK healthcare is the combined challenge of **limited procurement expertise** within NHS fragmented procurement efforts across provider organizations. Procuring AI requires complex costly due diligence, including market scanning, assessing technical fit, validating tools, and evaluating real-world performance, all of which demand time, resources, and technical skills. Many provider organizations lack the capacity to assess AI solutions independently, particularly given additional clinical and information governance obligations. Traditional arms-length procurement models - where the buyer and supplier maintain a formal, independent, and transactional relationship – add to this complexity, by limiting early testing, feedback, and shared learning between suppliers and adopters. This combination of factors frequently leads to suboptimal procurement decisions, delayed deployments, and limited impact of AI innovations.

“Everywhere that thinks about purchasing it and then adopting it, then feels like they want to do their own local evaluation. And indeed, that might be the right thing to do, but it means that the work that was done with them [the supplier], with another ward is much less valuable… The commercial companies are very frustrated by this because they don't want to have to redo the evaluation in every center…” (Adopter)

Integrating AI into routine healthcare practice is inherently challenging—not only because of the technical demands but because it necessitates **rethinking how services are designed and delivered**. As one interviewee observed, AI should not be a “sticking plaster” for poorly organized systems; rather, its real value lies in prompting much-needed transformation.

“And by that and I'm thinking about not only the AI implementation, but also the transformation of the underlying service… my view is that if AI a sticking plaster to a badly organized and designed service or process; It is just the wrong approach… In fact, I go as far as to say that I think half the benefit of AI driven transformation is just the fact that it initiates transformation.” (Adopter)

The **differences in digital maturity are compounded by the variations in the availability and quality of data across adopting sites**. AI systems require vast amounts of high-quality data to be developed, validated, and to function effectively, but many healthcare organizations face data silos, poor data quality, and limited access to relevant datasets. This ‘feeding the beast’ problem constrains AI’s potential and hampers efforts to scale solutions beyond initial pilot settings.

“And I'm fairly certain in cancer and kidney there was little to no data available, so [system_name] predictor was not being tried in those clinics” (Supplier)

Integrating individual clinical applications with specific hospital infrastructures is often expensive and inefficient, leading to duplication of effort and fragmentation. However, the implementation efforts so far have been focused on local adoption of AI applications. The **absence of shared digital platforms** and interoperable systems prevents AI from being effectively integrated across different care settings, limiting their scalability and impact. The NHS AI lab has attempted to create shared infrastructures across multiple trusts, but has faced technical and legal challenges.

### Market and incentive misalignment

The fourth mechanism relates to tensions between the economic realities of AI innovation and the structure of healthcare markets. Suppliers described significant uncertainty surrounding return on investment, routes to market, partnership arrangements, reimbursement, and long-term commercial viability. At the same time, healthcare organizations faced competing pressures around risk, affordability, procurement, and evidence requirements. These conditions created misaligned incentives between innovators, adopters, regulators, and funders. Stakeholders frequently described situations in which technically promising innovations struggled to secure investment, generate the evidence required for adoption, or navigate complex healthcare purchasing environments, limiting their ability to scale sustainably. As highlighted in previous sections, the relationship between the healthcare organizations, AI vendors, and other commercial players is often unclear. Vendors face challenges in **navigating partnerships while maintaining fair competition** and meeting broader socio-economic objectives, such as equity, accessibility, and public benefit. The absence of a structured framework for collaboration, and the arms-length procurement models between suppliers and adopters, can lead to inefficiencies and misaligned expectations between different stakeholders. The **lack of clear ROI** models for AI in healthcare discourages investment and scaling efforts. Many vendors, especially those developing AI for the NHS for the first time, find it difficult to demonstrate the financial and operational benefits of their AI solutions in a way that aligns with NHS priorities. This uncertainty can make investors hesitant to back AI companies targeting the healthcare sector, slowing down commercialization efforts. AI vendors often face misalignment between their objectives and those of funders and healthcare adopters. While public funders may prioritize innovation and research, healthcare providers focus on operational efficiency and patient outcomes. This disconnect can lead to difficulties for technology vendors in securing long-term funding, integrating AI into clinical workflows, and achieving widespread adoption.

“So financially we haven't really seen a huge benefit just because of the kind of restrictions that were placed on us as a factor. You couldn't go to market until that commissioned agreement was signed. It is still in motion.” (Supplier)

“[After the pilot phase] we felt it was a shame to switch it off at the end of the project without knowing whether It would be of interest to them in the future if there was a commercialized model. So actually we've continued to offer it to those trusts for free is my understanding on the on the provision that they kinda continue to work with us as we do make some iterations to the product and enhance the product and give us feedback and do some testing for us.” (Supplier)

AI innovators, both start-ups and established international firms, also pointed to the **struggles to navigate the fragmented and complex healthcare landscape**. Our observation shows, multiple points of access to the system are possible, each with different requirements, funding mechanisms, and approval processes. Some pathways are well-established, while others are emerging without clear guidance, making it difficult for businesses to determine the most effective route to market. Additionally there are multiple control points and multiple stakeholders, including clinicians, administrators, procurement officers, and policymakers within the health system. Due to this multiplicity, decision-making processes are fragmented, and approval pathways vary across different NHS trusts and regional bodies. This complexity makes it difficult for AI vendors to navigate the system and scale their solutions efficiently.

Table 5 provides an overview of the 15 challenges identified through the analysis and their mapping to the four higher-order mechanisms.

**Table 5.** System-level mechanisms that constrain the scaling of AI.

System-level mechanism	Associated challenges (Original 15)	Interpretation (analytical link)
M1. Fragmented and project-based innovation pathways (“pilot-to-scale gap”)	• Unclear pathway from pilot implementation to widespread adoption • Limited focus on funding AI scaling • Unclear evaluation approaches for changing AI products • Unclear mechanisms to systematically assess and approve AI adoption • Long lag time between product launch and income	Innovation is structured around short-term pilots rather than sustained pathways, leading to discontinuity between experimentation and adoption. Weak evaluation and funding structures further delay or prevent scaling.
M2. Fragmented governance and lack of system orchestration	• Lack of system-view of change • Poor alignment between regulatory processes and the dynamics of AI innovation • Differences across adopting countries (roles, healthcare funding, etc.) • Broader economic and political drivers	Misalignment across policy, regulatory, and institutional structures limits coordinated action and shared direction, resulting in fragmented decision-making and inconsistent scaling conditions.
M3. Organizational transformation deficit	• Limited procurement expertise by healthcare provider organizations • Need for rethinking healthcare service design and delivery • Differences in levels of digital and data maturity	Healthcare organizations lack the capabilities, infrastructure, and readiness required to integrate AI into workflows and redesign services, constraining adoption beyond isolated use cases.
M4. Market and incentive misalignment	• Unclear partnership models • Lack of clear return on investment • Struggles to navigate the fragmented and complex healthcare landscape	Misaligned incentives between suppliers and adopters, combined with fragmented market structures, create uncertainty and reduce the viability of scaling AI solutions.

## Discussion

### Summary of findings and contribution to literature

Several recent reviews have catalogued various challenges to AI adoption in healthcare, commonly highlighting issues such as trust, ethics, workflow disruption, workforce capability, data quality, and evaluation.^17,18^ Many studies also identify technical and data challenges such as algorithmic bias from homogeneous datasets and interoperability with existing infrastructures as barriers in large scale adoption of AI in the sector.^18–20^ Regulatory issues are also specified as key challenges to moving from clinical trials to large scale adoption of AI.^9,18–20^ While these studies provide an important foundation, they often treat challenges as discrete factors or organize them by domain. Our contribution differs in two ways. First, drawing on a large empirical dataset across interviews, observations, and documents, we show that these challenges are not best understood as isolated obstacles but as interdependent system-level mechanisms. Second, we focus specifically on the transition from pilot experimentation to wider adoption, an area that remains comparatively underdeveloped in much of the AI implementation literature.

Our analysis suggests that the challenges identified in prior work are better understood as manifestations of four mutually reinforcing mechanisms: fragmented and project-based innovation pathways, fragmented governance and lack of system orchestration, organizational transformation deficits, and market and incentive misalignment. These mechanisms are analytically distinct because they describe different system-level dynamics rather than different categories of barriers. *Fragmented and project-based innovation pathways* capture discontinuities between experimentation and routine adoption; *fragmented governance and lack of system orchestration* describe coordination failures across institutions responsible for innovation, regulation, procurement, and implementation; *organizational transformation deficits* refer to limitations in the capabilities required to redesign services and workflows around AI; and *market and incentive misalignment* reflects tensions between the economic incentives of suppliers, adopters, and funders. Together, these mechanisms provide a higher-order explanation of why challenges that appear separate in practice repeatedly co-occur and reinforce one another. This is the main analytical advancement of the paper. The novelty does not lie in claiming that procurement, regulation, infrastructure, or evaluation are newly discovered challenges; rather, it lies in showing how these challenges cluster into a small number of system dynamics that together explain why scaling remains difficult despite sustained policy attention and investment. In this sense, the paper moves from a descriptive account of “what the barriers are” to a system-level explanation of why they persist and how they reinforce one another. For example, fragmented governance and regulatory uncertainty can contribute to unclear partnership models and weak routes to procurement, while these in turn amplify organizational capability gaps and reduce suppliers’ confidence that investment in scaling will be recoverable.^11^ It is important to note that the identified mechanisms are not intended as deterministic causal explanations, but as analytically derived system-level dynamics that help interpret why barriers to scaling recur and reinforce one another across contexts and stakeholder groups.

This finding extends existing implementation frameworks, including Non-adoption, Abandonment, Scale-up, Spread, and Sustainability (NASSS).^21^ The NASSS framework provides an influential way of understanding complexity across domains such as the technology, value proposition, adopter system, organization, wider system, and adaptation over time. Importantly, our contribution is not simply the identification of additional factors or a broader system perspective. Rather, the mechanisms identified here explain how challenges located across different NASSS domains become linked through recurring system dynamics. In this sense, the paper moves beyond categorizing complexity to explaining how complexity is reproduced over time and why promising AI innovations repeatedly fail to progress beyond pilot implementation. First, it identifies cross-domain mechanisms rather than remaining at the level of domains or factors. Second, it shows the feedback loops between these mechanisms -for example, how weaknesses in the wider system shape the value proposition for suppliers and the implementation capacity of provider organizations. The central contribution of the paper is therefore an empirically grounded explanation of the pilot-to-scale gap. Rather than treating scaling as a continuation of implementation, our findings suggest that movement from pilot experimentation to routine deployment is shaped by a distinct set of system-level dynamics involving governance, markets, organizational transformation, and innovation pathways. Third, it foregrounds the structural problem of the pilot-to-scale gap, showing that the failure to build coherent pathways from experimentation to routine service integration is not just an implementation problem inside organizations, but a system property reinforced through governance, market, and organizational interactions. This point also resonates with Hughes et al.’s^22^ Service Readiness Level framework, which emphasizes that scaling depends on the accumulation of different forms of evidence over time; our findings suggest that evidence readiness alone is insufficient unless accompanied by aligned governance, procurement, funding, and partnership arrangements.

At the same time, greater system-level coordination should not be assumed to be an unqualified solution. While fragmentation creates significant barriers to scaling, excessive centralization may introduce new challenges, including bureaucratic inertia, reduced opportunities for local experimentation, over-centralized procurement processes, and increased risks of vendor lock-in. The challenge is therefore not simply to coordinate more, but to develop governance arrangements that balance system-wide alignment with local flexibility, learning, and innovation.

Our study also contributes to recent empirical studies of AI implementation and scaling. Work in radiology has shown that implementation is shaped not only by the technical performance of AI systems, but by how deployment strategies are organized, how technologies are anchored in local practice, and how deployment processes are coordinated. Severinsen et al.^23^ how that both top-down and bottom-up implementation approaches can generate useful learning, but also imply different forms of coordination and reuse. Ellingsen et al.^24^ argue that AI projects in radiology can become detached from local practice when scaling ambitions outpace local anchoring. Silsand et al.^25^ similarly demonstrate that real-world deployment requires attention to the practical work of integration, adaptation, and evaluation in context. Our findings converge with these studies, but extend them by shifting the analytical focus beyond individual deployment processes or specialty settings to the broader system conditions that shape whether promising pilots can progress into routinised and sustainable adoption across organizations. Put differently, whereas much of the recent literature has examined how organizations implement AI, our study explains why the broader ecosystem so often fails to convert local success into system-wide scaling.

Our study also contributes to studies of market dynamics for AI scaling. A recent study by Adler-Milstein et al.^26^ argues that third-party AI developers compete in markets strongly shaped by incumbent Electronic Health Record (EHR) vendors, whose infrastructural position gives them major advantages in adoption. Dominant US-based radiology services companies are also acquiring promising app developers to create comprehensive AI-enabled radiology offerings. This reinforces our finding that AI scaling cannot be explained only through organizational readiness or clinician attitudes. Supplier incentives, platform dependencies, procurement pathways, and the distribution of market power shape which tools survive long enough to scale and which do not. Our emphasis on market and incentive misalignment therefore adds an important dimension to implementation debates that often concentrate more heavily on adoption within provider organizations than on the commercial environment in which suppliers operate.

Moreover, many of the challenges identified in this study are not unique to AI and have been seen in broader digital transformation initiatives in the health sector, including issues of procurement, organizational readiness, interoperability, and governance. However, stakeholders also highlighted challenges that are particularly pronounced in the context of AI, including evolving regulatory requirements, difficulties evaluating adaptive and continuously changing technologies, uncertainty regarding accountability for algorithmic decisions, and the need to generate evidence while technologies continue to evolve. These characteristics amplify existing implementation challenges and contribute to the persistence of the pilot-to-scale gap.

### Strategic approaches to addressing challenges to scaling artificial intelligence

Building on the four systemic mechanisms identified in the Results, and combining them with the extant literature, our analysis suggests that addressing challenges to AI scaling requires a set of coordinated strategic responses. Each strategy targets specific underlying system dynamics while also addressing their interdependencies. Rather than independent solutions, these approaches operate as complementary interventions across governance, organizational transformation, market design, and evaluation infrastructures. Table 6 maps the strategic responses to the identified mechanisms and their underlying empirical challenges.

**Table 6.** Strategic approaches to addressing challenges to scaling artificial intelligence.

Mechanism	Core scaling problem	Strategic implication
M1: Pilot-to-Scale Gap	No pathway from pilot to adoption	Coherent pathways (funding, regulation, partnerships) + Continuous evaluation
M2: Fragmented Governance	Lack of coordination across actors	From Silos to Synergy (concerted adoption, system orchestration)
M3: Transformation Deficit	AI used for automation, not redesign	Triple Imperative (automation–augmentation–transformation)
M4: Market Misalignment	Incentives and ROI unclear	Coherent pathways (market shaping, procurement, funding reform)

The strategic approaches presented in Table 6 should not be interpreted as direct empirical findings. Rather, they represent an interpretive synthesis developed through engagement between the empirical findings and existing literature on innovation, implementation, and digital transformation. Specifically, the strategies build upon the identified system-level mechanisms and the recurring challenges raised across stakeholder groups. While some elements represent direct empirical insights (e.g., fragmented pathways, and procurement challenges), others are interpretative extensions informed by the literature and our system-level analysis. Together, the strategies provide a bridge between empirical explanation and action, combining empirically grounded observations with theoretically informed and normative recommendations for addressing AI scaling challenges.

### From silos to synergy

This strategy addresses the fragmented governance and lack of system orchestration (Mechanism 2) identified in our analysis, by promoting coordinated, cross-organizational approaches to innovation and scaling.

Studies show that AI development in healthcare is still largely limited to isolated applications or narrowly focused pilot projects.^27^ While bottom-up exploration and innovation remain important, our study shows that scaling AI in healthcare requires a more integrated, system-wide approach that supports ongoing learning across organizations and diverse players, enabling broad dissemination, and long-term impact. Cresswell, Sullivan^28^ refers to this as concerted adoption which involves coordinated and collective effort by multiple stakeholders such as policymakers, healthcare professionals, IT vendors, managers, and patients. Addressing the challenges highlighted in our study call for approaches that support collaborative innovation across organizations^29^ and encourage stronger networks to enable transformation at scale. This cross-organizational innovation and collaborative learning across different networks paves the way for a shared sense of direction and alignment as well as system-wide integration of innovations, that can develop over time to enable scaling beyond individual organizations and pilot sites.^13,30^ This approach encourages a more comprehensive attempt to innovation that offers a pathway toward more connected and scalable solutions - one that connects technical invention with value creation, stakeholder alignment, and sustained impact.^31^ In order to do this, there is a need to rethink leadership and governance structures across organizational boundaries. Governance must go beyond compliance or project oversight; it must create the enabling conditions for distributed decision-making, risk-sharing, and shared learning, as well as creating clear pathways for growth.

Furthermore, integrating individual clinical applications with specific hospital infrastructures is often expensive and inefficient, leading to duplication of effort and fragmented solutions.^32^ These challenges are driving a shift toward platform-based models^33^ for deploying clinical applications, offering a more scalable and cost-effective approach to AI adoption.

### The triple imperative of automation, augmentation, and transformation

This strategy responds to the organizational transformation deficit (Mechanism 3), where AI adoption remains focused on automation rather than enabling deeper service and system re-design.

Key challenges identified in our research included the absence of a holistic perspective on change. Instead of aiming for broader service or system-wide transformations, the focus was often on automating current tasks within existing workflows and regulatory structures. This approach led to solutions that were narrowly focused on specific use cases, limiting their ability to scale. While this pattern is sometimes interpreted as a lack of ambition, our findings suggest that it is also shaped by system-level constraints, including fragmented governance, regulatory and procurement complexity, and misaligned incentives, which tend to favor incremental, lower-risk innovations over more disruptive forms of change.

The emphasis on AI’s technological capabilities often overshadowed the more foundational organizational changes required to fully realize its benefits. However, effective adoption and scaling of AI requires a deliberate balance between automation, augmentation, and transformation.

Automation typically targets discrete, repetitive tasks, aiming to improve efficiency and reduce human workload - for example, streamlining administrative functions or automating image recognition in diagnostics . By contrast, transformation represents the most disruptive and ambitious application of AI. It involves rethinking and redesigning care pathways, workflows, and even institutional goals, in response to the new capabilities AI enables.^34^ Yet this requires organizations to manage deep cultural and operational shifts. In large, highly regulated healthcare systems, such shifts can be difficult to realize, as institutional pressures and existing organizational structures often reinforce continuity and incremental adaptation rather than radical change.

Situated between automation and transformation is augmentation - a form of AI adoption that enhances rather than replaces human decision-making. Augmentation involves using AI tools to support human decision-making – such as in reading radiology results - thereby enriching clinical judgment and supporting more informed, timely decisions. More advanced systems can synthesis complex data, identify patterns, and offer evidence-based recommendations to clinicians, supporting them in interpreting information and improving the quality of care.^35^ Importantly, augmentation often occurs without altering existing structures or workflows; it embeds AI within current practices in a way that enhances human expertise but does not yet push toward wider organizational or systemic change. In this sense, augmentation is an incremental step: it improves practice through the addition of novel analytical capabilities, but it typically does not challenge the underlying design of services.

While many of the cases we studied involved AI systems that automated or augmented existing clinical and administrative tasks within individual healthcare organizations, few had advanced toward a transformative use of AI. This reflects not only the early-stage nature of many projects, but also the broader system dynamics identified in this study, which make large-scale transformation difficult to achieve. The relatively short duration of the Lab’s interventions further limited the opportunity to optimize its use and explore the more disruptive and systemic changes that AI might enable. Yet this is where the full potential of AI lies: in supporting new models of care such as proactive, personalized interventions, remote diagnostics often utilizing patient-generated data, and integrated population health management that transcend the boundaries of traditional institutions.

Realizing such transformative potential requires more than isolated pilot projects or local experimentation. It demands coordinated, system-level change supported by national strategies, coherent regulatory guidance, shared learning, sustained investment, and shared digital infrastructure. Rather than simply implementing new tools, healthcare leaders must rethink roles, processes, and objectives and service re-design. This also requires moving beyond the dominant view of transforming single organizations to transformation at system and sector level.^13^ Only with such comprehensive, policy-backed frameworks can we align incentives, ensure interoperability, manage emerging risks, and support equitable access to AI-enabled services across diverse care settings and populations.

### Building coherent pathways through regulation, partnership and funding

This strategy addresses both the pilot-to-scale gap (Mechanism 1) and market misalignment (Mechanism 4), by creating clearer and more sustainable pathways for innovation to move from experimentation to system-wide adoption.

Many vendors face an unclear path from pilot to scale. As noted by Klein, Neitzert^36^ a lack of commercialization clarity often leads to challenges to startups including such as innovation fatigue, disengagement from healthcare adopters, or even death. A more coherent regulatory and investment ecosystem is essential, combining clearer evidence requirements, harmonized funding priorities, and structured partnership frameworks. National AI strategies must therefore bridge the gap between innovation funding and operational sustainability,^31^ ensuring that policy supports not just invention, but adoption and scale.

The challenges identified in this research compound the already high failure rates among start-ups. Many AI-driven healthcare companies struggle to survive due to long approval-to-reimbursement timelines, which often exhaust financial resources before revenue generation is possible. Our findings suggest that, in the context of regulatory and procurement bottlenecks, widely recognized as barriers to AI adoption in the NHS,^37^ and uneven investment dynamics across the innovation pathway,^38^ some start-ups initially supported by the NHS AI Lab may face constraints in scaling independently. In such conditions, acquisition by larger (often international) firms can represent a pragmatic route to sustainability. While this may support short-term viability, it also raises potential concerns regarding the retention of UK-developed intellectual property and longer-term strategic value, particularly given broader challenges in translating innovation into system-wide impact. Market dynamics further complicate AI scaling in healthcare. While entrepreneurs recognize the substantial risks associated with entering the field, the allure of a large potential market and an expanding ecosystem of AI-driven innovation continues to attract new entrants. Yet, the highly professionalized nature of healthcare, coupled with the attitudes of key stakeholders - particularly clinicians and consultants - toward technological innovation, and ambiguity of the macro-environment, creates challenges that often result in promising initiatives reaching dead ends rather than widespread adoption. These challenges call into question the relevance of traditional technology adoption models, such as Geoffrey Moore’s “Crossing the Chasm”.^39^ While early adopters play a critical role in validating AI solutions, their influence may be insufficient to drive broader adoption within healthcare due to the sector’s regulatory constraints, complex systems of entry and growth, and professional norms. Instead, bridging the gap to the early majority, who require demonstrable reliability and clear benefits, may be the key to overcoming barriers to AI scaling in healthcare and therefore fundings are needed to bridge this gap. Furthermore, new procurement models like the EU competitive dialogue-based procurement process may be helpful here.^40^

National regulatory strategies should shift from precautionary models to dynamic, cyclical ongoing risk governance approaches.^41^ This is particularly important given that, under frameworks such as the EU AI Act, many healthcare AI applications are classified as high risk, requiring continuous monitoring and oversight. These frameworks must be adaptable to address the challenges posed by AI, integrating innovation with strong oversight to ensure patient safety without discouraging market entry. By fostering innovation within both new and established companies, regulations can balance public health protection and market growth. Well-designed, flexible regulations not only benefit healthcare systems but also support economic growth by creating an environment conducive to business development. A collaborative regulatory approach can align the interests of regulators, innovators, and the public, ultimately benefiting society and the economy.

### Continuous formative evaluation and post-market surveillance

This strategy targets the pilot-to-scale gap (Mechanism 1) by embedding ongoing learning and adaptive evaluation across the AI lifecycle.

Scaling AI is not a single-stage event but a dynamic and iterative process that requires continuous evaluation from development to deployment and beyond. While post-market surveillance - monitoring performance of deployed AI systems -^42^ is essential to detect unintended consequences, performance drifts, or adverse outcomes, it is equally important to integrate formative evaluation during earlier phases such as development, adoption, implementation, and optimization.

Formative evaluation helps organizations and developers assess the relevance, feasibility, and impact of AI technologies in context, adapting tools and strategies as needs evolve. This ongoing evaluation allows stakeholders to address misalignments between AI design and practical use, fostering trust and usability from the outset.^43^ Without such feedback mechanisms, there is a risk that AI tools may fail to meet user expectations or institutional goals despite being technically sound.

Post-market surveillance^42^ complements this by ensuring long-term accountability and learning after deployment. Especially in high-stakes environments like healthcare, AI systems require continuous monitoring to detect emerging risks, shifts in performance, and changes in user behavior over time.^2,4^ Together, formative evaluation and post-market surveillance form a comprehensive governance framework that enables safe, adaptive, and responsible AI integration across its full lifecycle.

### A system-level model for scaling AI in healthcare

Building on the four interrelated system-level mechanisms identified - fragmented and project-based innovation pathways, fragmented governance and lack of system orchestration, organizational transformation deficits, and market and incentive misalignment - we proposed a set of complementary strategic responses that collectively address these underlying dynamics. These mechanisms do not operate independently; rather, they reinforce one another, creating a self-sustaining cycle that constrains the transition from pilot experimentation to system-wide adoption. Accordingly, effective scaling requires coordinated interventions that target multiple points in the system simultaneously. The shift from silos to synergy responds to fragmented governance by promoting cross-organizational coordination, shared learning, and distributed decision-making. The triple imperative of automation, augmentation, and transformation addresses organizational transformation deficits by encouraging healthcare systems to move beyond incremental efficiency gains toward deeper service redesign. The development of coherent pathways through aligned regulation, funding, and partnership models tackles both the pilot-to-scale gap and market misalignment by creating clearer and more sustainable routes from innovation to adoption. Finally, continuous formative evaluation and post-market surveillance strengthen these pathways by embedding iterative learning and adaptive governance across the AI lifecycle. Taken together, these strategies function as an integrated response to interacting system constraints, have the potential to reduce some of the fragmented, pilot-driven innovation in order to move toward sustainable, system-wide scaling of AI in healthcare.

**Figure 1.**
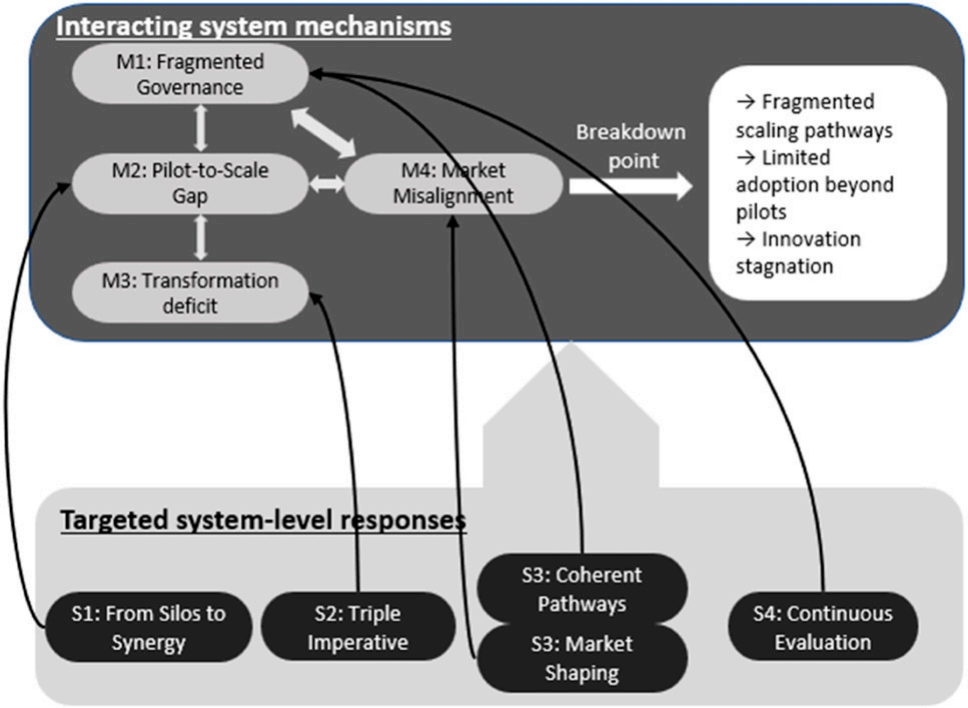
Interpretive system-level model of mechanisms constraining AI Scaling in healthcare and corresponding strategic responses (Arrows indicate interrelationships and reinforcing dynamics between mechanisms and strategic responses).

Figure 1 presents a system-level model explaining the persistent challenges in scaling AI in healthcare and the corresponding strategic responses required to address them. The upper panel illustrates four interrelated mechanisms: (1) fragmented and project-based innovation pathways (“pilot-to-scale gap”), (2) fragmented governance and lack of system orchestration, (3) organizational transformation deficits, and (4) market and incentive misalignment, which collectively constrain the transition from pilot experimentation to system-wide adoption. The lower panel outlines targeted system-level responses that address these underlying dynamics, including coordinated cross-organizational approaches, organizational transformation, coherent regulatory and funding pathways, and continuous evaluation across the AI lifecycle.

### Implications for policy and practice

The findings of this study suggest that scaling artificial intelligence (AI) in healthcare cannot be achieved through isolated interventions targeting individual challenges. Rather, the persistence of challenges reflects a set of interdependent system-level mechanisms that operate across policy, organizational, and market domains. As such, effective responses must be coordinated, multi-level, and aligned with these underlying dynamics.

**From Pilot-Driven Innovation to System-Level Pathways:** A central implication concerns the need to move beyond pilot-driven innovation toward the development of coherent pathways for scaling. The widespread absence of clear routes from experimentation to adoption reflects a structural “pilot-to-scale gap,” where promising innovations fail to translate into routine practice. Addressing this requires policy frameworks that link innovation funding to long-term value creation, including provisions for implementation, integration, and ongoing evaluation. Early-stage innovation programs should therefore incorporate explicit planning for procurement, integration into existing systems, and sustainability, rather than treating these as downstream considerations.

**Strengthening System-Level Governance and Coordination:** The fragmentation of governance across regulatory, funding, and organizational domains presents a major barrier to scaling. This highlights the need for stronger system-level coordination and orchestration mechanisms, including the role of dedicated intermediaries or national bodies that can align stakeholders, offer new evaluation approaches, and support navigation across complex innovation pathways. Rather than operating as isolated initiatives, AI programs should be embedded within coherent national strategies that integrate regulatory, financial, and operational dimensions.

**Supporting Organizational Transformation, Not Just Adoption:** Current approaches in AI adoption, often prioritize automation within existing workflows, reflecting both organizational constraints and wider system pressures that favor incremental change. However, realizing the full benefits of AI requires investment in capabilities for service redesign, including workforce training, process reconfiguration, and data infrastructure. Policy and organizational strategies should therefore move beyond technology deployment to support socio-technical transformation, ensuring that AI is integrated in ways that reshape, rather than merely reinforce, existing practices.

**Aligning Market Incentives and Commercial Pathways:** Unclear commercialization pathways, long timeframes to revenue, and fragmented procurement processes create uncertainty that discourages sustained investment. Addressing this requires clearer and more consistent routes from pilot to scale, including aligned regulatory requirements, procurement mechanisms, and funding models. Greater transparency in evaluation standards and decision-making processes can also support more effective partnerships between vendors and healthcare organizations, reducing friction and enabling more sustainable scaling trajectories.

**Enabling Networked Learning and Cross-Organizational Collaboration:** Finally, the findings point to the importance of shared learning and innovation. Scaling AI requires not only local implementation but also the ability to diffuse knowledge, evidence, and practices across organizational boundaries. This includes developing platforms and repositories for sharing implementation experiences, evaluation outcomes, and reusable components. More broadly, fostering collaborative ecosystems which includes linking providers, suppliers, researchers, and policymakers can support coordinated adoption and reduce fragmentation. Such approaches are particularly important in complex healthcare systems, where scaling depends on alignment across multiple stakeholders and institutions.

### Strengths and limitations

The key strength of our paper is that our findings show that scaling AI in healthcare is not primarily a matter of overcoming isolated technical or organizational challenges. It requires simultaneous attention to innovation pathways, governance arrangements, organizational transformation, and market design. This is why the strategic responses proposed in this paper are intentionally coordinated rather than standalone. The persistence of challenges despite substantial investment reflects not a shortage of promising pilots, but the failure to address multiple reinforcing mechanisms at the same time. Seen in this way, AI scaling is best understood as a problem of system alignment and orchestration, not simply one of technology adoption.

The transferability of our findings requires careful qualification. The NHS England has distinctive features, including its national policy architecture, publicly funded and highly regulated service environment, fragmented local procurement arrangements, and uneven digital maturity across organizations. These features shape the particular form taken by the challenges we observed. Our findings should therefore not be read as universally generalizable in a simple sense. Rather, we suggest that the mechanisms identified here are likely to be most transferable to health systems that share some combination of strong public regulation, complex multi-organizational care delivery, and dependence on public procurement for scaling digital innovation. In settings with different reimbursement systems, vendor markets, or governance arrangements, the same mechanisms may appear in different forms or with different intensity. This is consistent with comparative work showing that AI implementation pathways vary according to how health systems organize governance, funding, and innovation support. The value of the model, therefore, lies less in claiming universal sameness and more in offering a framework for analyzing how local institutional arrangements shape the transition from pilot to scale. Additionally while our focus on more mature pilots enabled deeper analysis of scaling processes, it may underrepresent challenges affecting early-stage or unsuccessful innovations.

A further limitation relates to sampling and reflexivity. The initial stakeholder list was provided by AI Lab leadership, which may have shaped early access to participants and introduced potential selection bias. Although we mitigated this through snowball sampling, the research team’s networks, and the inclusion of diverse stakeholder groups, some perspectives may have remained underrepresented.

## Conclusion

Without system-wide interventions and structural improvements, the large-scale deployment of AI in healthcare is likely to remain slow and fragmented. Our findings suggest that this is not simply due to isolated challenges, but to a set of interdependent system-level mechanisms that constrain the transition from pilot experimentation to widespread adoption. Addressing these requires coordinated action across policy, organizational, and market domains, including- encompassing navigating cross-organizational collaborations and supporting broader system transformations.

The centralized nature of the NHS provides a significant opportunity to consolidate efforts. Future comparative work of international strategies is vital to ensure that lessons surrounding scaling are shared. By establishing dedicated AI evaluation bodies, streamlining funding processes, and fostering transparent vendor partnerships, healthcare systems can support scalability and sustainability of AI adoption, with the aim of improving patient outcomes and operational efficiencies.(Supplemental material)

## Supplemental material

Supplemental material - A system-level analysis of challenges and strategies for scaling artificial intelligence in healthcare: A qualitative study of the NHS AI lab Supplemental material for A system-level analysis of challenges and strategies for scaling artificial intelligence in healthcare: A qualitative study of the NHS AI lab by Hajar Mozaffar, Robin Williams, Stuart Anderson, Kathrin Cresswell in DIGITAL HEALTH

## Data Availability

Data can be partially available on request due to ethical considerations.*
