# Supplementary material for: A system-level analysis of challenges and strategies for scaling artificial intelligence in healthcare: A qualitative study of the NHS AI lab
Source: Digit Health. 2026 Aug 10;12:20552076261464270. doi: 10.1177/20552076261464270 (PMC13458137; doi:10.1177/20552076261464270)
Supplement: Supplementary file 1 [file sj-pdf-1-dhj-10.1177_20552076261464270.pdf]

Interview topic guide

Please note that these questions will be tailored to participants' roles. These include:

- early adopter of AI systems or initiatives included in the AI Lab (healthcare professional, researcher, or implementer)
- system or technology developer
- involved in some other way in AI strategy in health and care

The areas to cover will also be tailored to emerging themes and in line with participants' perceived issues of importance. Key indicative areas to cover include:

- What has been your involvement in the AI Lab?
- What are your opinions of the AI Lab as an initiative?
  - Initial vision and to what extend this has been realised
  - Expected and realised benefits - prompts
    - Impact on safety, quality, efficiency of care;
    - Impact on development processes of AI
    - Impact on implementation of technology into practice
    - Impact on regulations and policies
    - Impact on market development
    - Value for money
    - Any other types of impact
  - Challenges and potential ways to address these
  - Differences between AI Lab components?
- Experience with AI Projects / Pilots
  - Have you been involved in a specific AI project and can you describe it - prompts
    - What problem was it trying to address?
    - What stage was it at?
  - What worked well in this project?
  - What challenges did you encounter - prompts
    - What happens after a pilot ends?
    - Pathways to move from pilot to wider adoption
    - Any difficulties to scale beyond initial trials?
    - Preparedness of healthcare organisations for adoption
    - Policy, regulation, or national strategies affecting AI scaling
    - Challenges of AI developers face in scaling solutions
  - What are the mechanisms used to over come challenges by different stakeholders?
- Lessons learned
  - How could impacts and value be maximised in the future?
  - What do you see as the biggest barriers to scaling AI in the next 5 years and how to deal with them?
  - Lessons learned and implications for future initiatives

Any other comments?

Any questions?
